# Correlation between periodontal disease management and metabolic control of type 2 diabetes mellitus. A systematic literature review

**DOI:** 10.4317/medoral.21048

**Published:** 2016-01-31

**Authors:** Flor-de-Liz Pérez-Losada, Enric Jané-Salas, María-del-Mar Sabater-Recolons, Albert Estrugo-Devesa, Juan-José Segura-Egea, José López-López

**Affiliations:** 1 DDS, Dentist. PhD student. Master of Oral Medicine, University of Barcelona, Spain; 2 PhD, DDS, MD. Professor of Oral Pathology. School of Dentistry, Barcelona University- Hospital Odontologíco Universidad de Barcelona. Oral Health and Masticatory System Group (Bellvitge Biomedical Research Institute) IDIBELL, University of Barcelona, L’Hospitalet de Llobregat, Barcelona, Spain; 3 PhD, DDS, MD. Professor of Oral Pathology. School of Dentistry, Barcelona University. Dentist at “Servei Catala de la Salut”, Spain; 4 PhD, DDS, MD. Professor of Endodontics. School of Dentistry, University of Seville, Spain

## Abstract

**Background:**

Diabetes and periodontal disease share common features in terms of inflammatory responses. Current scientific evidence suggests that treatment of periodontal disease might contribute to glycemic control. The objective of the study is a review of the last three years.

**Material and Methods:**

A literature search was performed in the MEDLINE (PubMed), Cochrane, and Scopus databases, for articles published between 01-01-2013 and 30-06-2015, applying the key terms “periodontal disease” AND “diabetes mellitus”. The review analyzed clinical trials of humans published in English and Spanish.

**Results:**

Thirteen clinical trials were reviewed, representing a total of 1,912 patients. Three of them had samples of <40 patients, making a total of 108 patients and the remaining ten samples had >40 patients, representing a total of 1,804. Only one article achieved a Jadad score of five. Seven articles (998 patients, 52.3% total), presented a statistically significant decrease in HbA1c (*p*< 0.05) as a result of periodontal treatment. In the six remaining articles (representing 914 patients, 47.8% of the total), the decrease in HbA1c was not significant. Patient follow-up varied between 3 to 12 months. In three articles, the follow-up was of 3, 4, and 9 months, in two 6 and 12 months.

**Conclusions:**

The majority of clinical trials showed that radicular curettage and smoothing, whether associated with antibiotics or not, can improve periodontal conditions in patients with diabetes mellitus. However, few studies suggest that this periodontal treatment improves metabolic control. However, there is no clear evidence of a relation between periodontal treatment and improved glycemic control in patients with type 2 diabetes mellitus.

**Key words:**Diabetes, periodontal disease, HbA1c, metabolic control.

## Introduction

Diabetes mellitus (DM) is a syndrome characterized by poor functioning of the carbohydrate, lipid and protein metabolism (1); its main characteristic is hyperglycemia (2). Hyperglycemia acts as the main cause of the progressive microvascular complications associated with diabetes (retinopathy, nephropathy, neuropathy) (3). There are various types of DM based on the complex interaction between genetic and environmental factors. Depending on the cause, factors that contribute to hyperglycemia can be: i) differences in insulin secretion; ii) reduction in glucose use; iii) increase in glucose production (4). DM is classified as two main types: Type 1 (DM1) and Type 2 (DM2). DM1 is associated with the destruction of β-pancreatic cells and it commonly appears in younger patients. DM2 is produced by a progressive decrease in sensitivity to insulin in its target tissues, and/or an insufficiency of the pancreas to increase insulin production, developing into a resistance to insulin. This type makes up 90-95% of all cases of diabetes (4). DM2 has been catalogued as a twenty-first century epidemic for both its frequency and impact in terms of cardiovascular disease and peripheral neuropathy. In recent years, it has been the main cause of death in the developed world (5,6); the prevalence of DM2 is very high in some western European countries (Germany, Spain, Italy, France, and the United Kingdom). In Spain, a recent institutional study has found a prevalence of 12.5% (6).

Age is an important risk factor for type DM2. In Europe, 37% of the population is aged over 50 years, and it is expected that this will increase to 44% by 2030 (6). For this reason, a dramatic increase in the numbers of diabetic patients is likely and foreseeable in the coming years (6).

DM diagnosis may be established through one of the following: i) Fasting blood glucose (≥ 8 hours without food) (FBG) ≥ 126 mg/dl (7.0 mmol/L); if the patient presents figures of between 100 and 125 mg/dl, then this points to pre-diabetes. ii) Blood glucose ≥ 200 mg/dl (11.1 mmol/L) two hours after oral glucose tolerance testing (OGTT). The test must be performed according to WHO guidelines established 1985, with 75 g anhydrous glucose dissolved in water after at least 8 hours without food. If the figures are between 149 and 199 mg/dl, this points to oral glucose intolerance. iii) If casual blood glucose is ≥ 200 mg/dl (11.1 mmol/L) (registered at any moment during the day, regardless of the time since food taken) and clinical DM symptoms are noted (poliuria, polidipsia, polifagia, unexplained weight loss) (4).

The glycosylated hemoglobin test (HbA1c) is considered the gold standard method for monitoring glycemia, which facilitates metabolic management of DM patients (7). The test measures mean glycemia during the last 2-3 months, allowing assessment of treatment efficacy and management by the patient him/herself (8).

Periodontal diseases comprise a group of pathologies characterized by periodontal inflammation caused by infection. In this way, they are induced by an accumulation of micro-organism, mainly bacteria, which provoke activation of the immune system to combat the infection. They start with gingival inflammation; if its etiological factors are not eliminated, the immune response will be more complex and the metabolism of the periodontal tissues will be upset, resulting in loss of periodontal support (9).

The inflammation involves an excessive production of inflammatory mediators, which end up causing tissue destruction. Among these, the most important are: interleukin 1β (IL-1β) and 6 (IL-6); prostaglandin E2 (PGE2); tumor necrosis factor alpha (TNFα), attaching to the receptor activator of nuclear factor kB (RANKL); matrix metalloproteinases (MMP); T cell regulatory cytokines (IL-12, IL-18); and chemokines (4,9). Inflammation is not a characteristic limited to periodontitis, but of many other diseases, including diabetes. In this way, diabetes is associated with high levels of systemic inflammatory markers (9).

The changes that diabetes can provoke in subgingival microbiota involve a greater prevalence of *Porphyromonas gingivalis* and *Prevotella intermedia* (10), the main bacteria involved in periodontal disease. Moreover, some research has cited DM as a risk factor for periodontal diseases (11,12), and among these, for apical periodontitis (13-15).

Current scientific evidence suggests that the treatment of periodontal disease might be capable of contributing to glycemic control. But the magnitude and clinical significance of this possible effect calls for better quality investigation. Several researchers have shown that radicular curettage and smoothing (RCS) can improve the periodontal state of patients with diabetes. But in relation to the metabolic control of diabetes, the results do not appear to be conclusive. While some studies have found that periodontal improvement is associated with better metabolic control (16-21), others have not identified any beneficial effect (22,23).

This systematic review examines clinical studies of humans that have investigated the possible correlation between periodontal treatment and glycemic control in patients with type 2 diabetes mellitus. The aim was to provide an answer to the clinical question: “Does periodontal treatment influence the metabolic management of DM2?” 

## Material and Methods

A search was performed in the MEDLINE (PubMed) database for articles published between 01-01-2013 and 30=06-2015 using the key search terms “Periodontal disease” AND “Diabetes mellitus”. The review included articles published in English and Spanish describing clinical studies of humans. Inclusion criteria were: i) a minimum sample size of 20 patients; ii) analytic data for HbA1c before and after periodontal intervention; iii) adequate description of non-surgical periodontal treatment (based on radicular curettage and smoothing); iv) a follow-up of at least two months. After the initial search and application of inclusion criteria, a further search was performed in the Cochrane and Scopus databases, discarding any repeats.

To evaluate the methodological quality of the clinical studies, levels of evidence and degrees of recommendation were examined following established guides to good clinical practice (24). The articles’ internal validity was measured using the Jadad scale (25). A score of 0-5 was assigned to each feature of the study, with a higher score indicating a higher quality of the contribution. 3-5 were considered good quality, 2 acceptable, and 0-1 of poor quality. The selection and review process was carried out by four reviewers (MS, EJ, JLL, FP)”

To collate data extracted from the studies, a specially drafted index card was prepared showing: author and year the study was carried out, evidence level according to Jadad scale (25), sample size, inclusion criteria, exclusion criteria, distribution of groups, characteristics of periodontal treatment performed, follow-up period, periodontal and systemic parameters registered, effects of periodontal treatment on periodontal state, effects of periodontal treatment on metabolic values. From these index cards, the most relevant data were collated in a comparison table.

## Results

**Figure 1:**
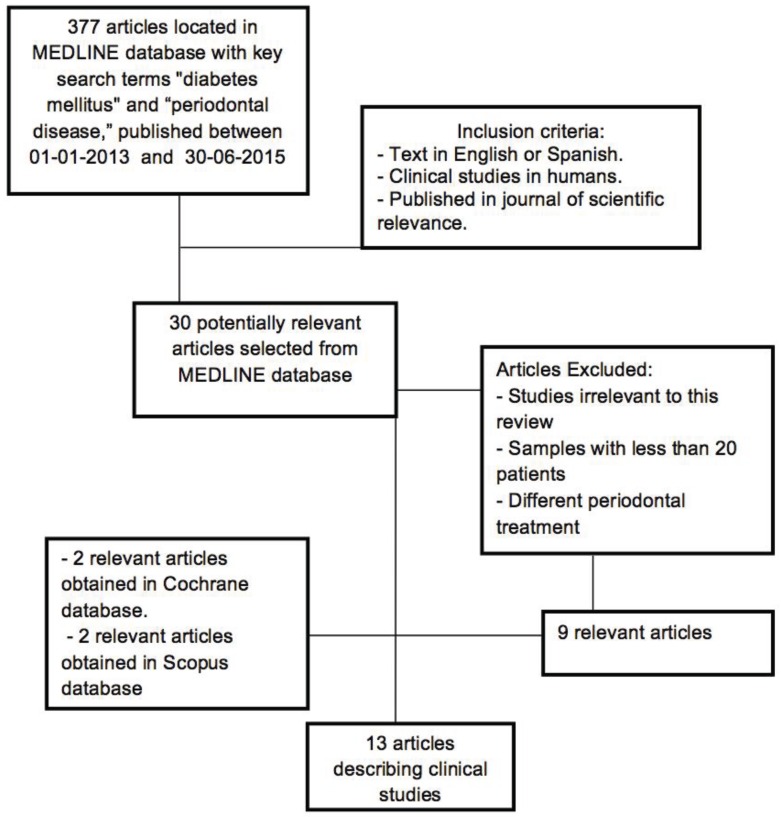
Flow diagram of selection process.

The initial search in the Medline database identified 377 articles of which 30 clinical trials in humans were selected. After applying the inclusion criteria this number reduced to nine (26-34). Searches in other databases (Cochrane and Scopus) obtained a total of 127 articles; when repetitions had been excluded and inclusion criteria applied, a further four articles (35-38) were added, making a total of 13 (Fig. 1) ( Table 1 and  Table 1continue). 

**Table 1:**
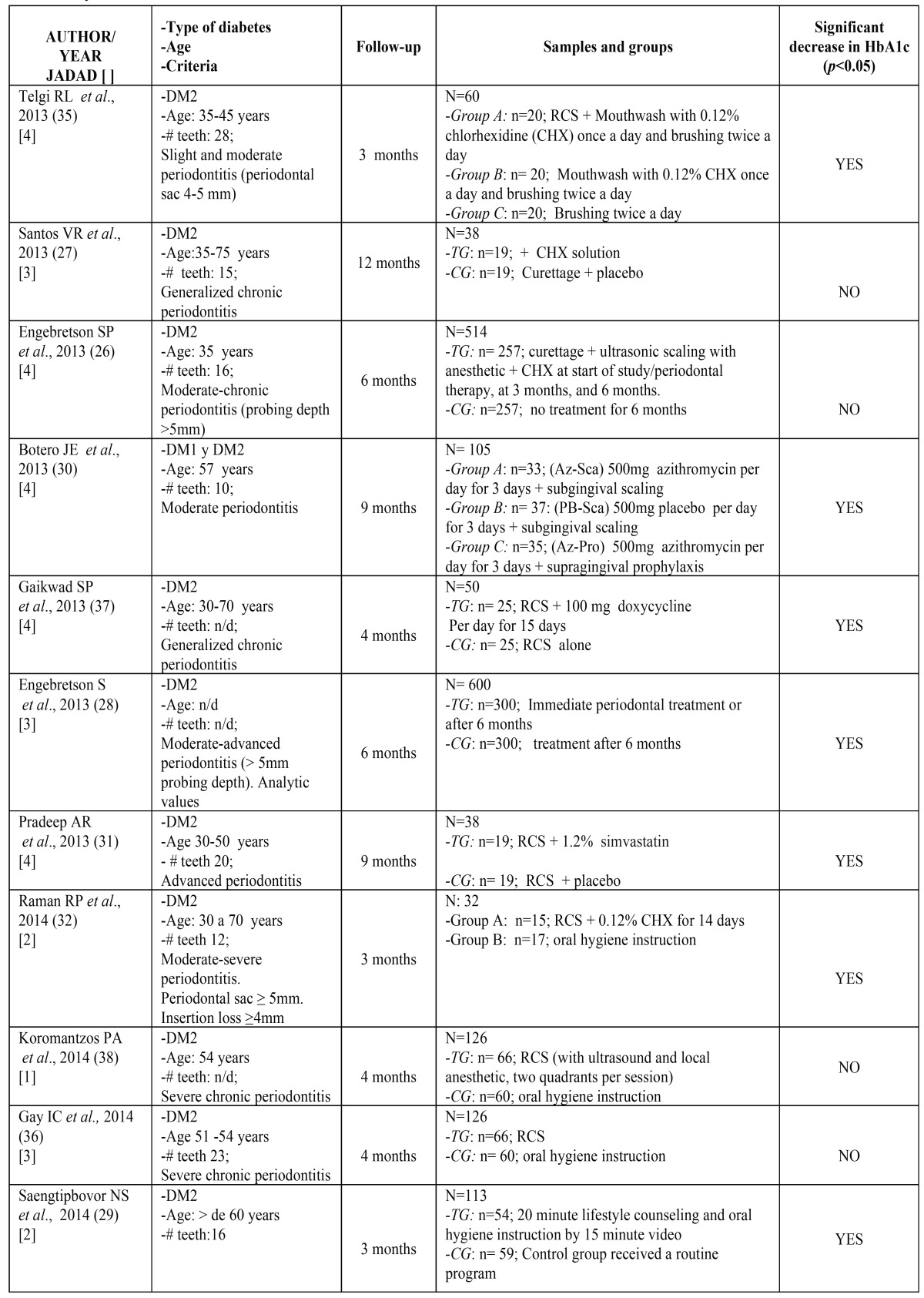
Key characteristics of the studies reviewed.

**Table 1 Continue:**
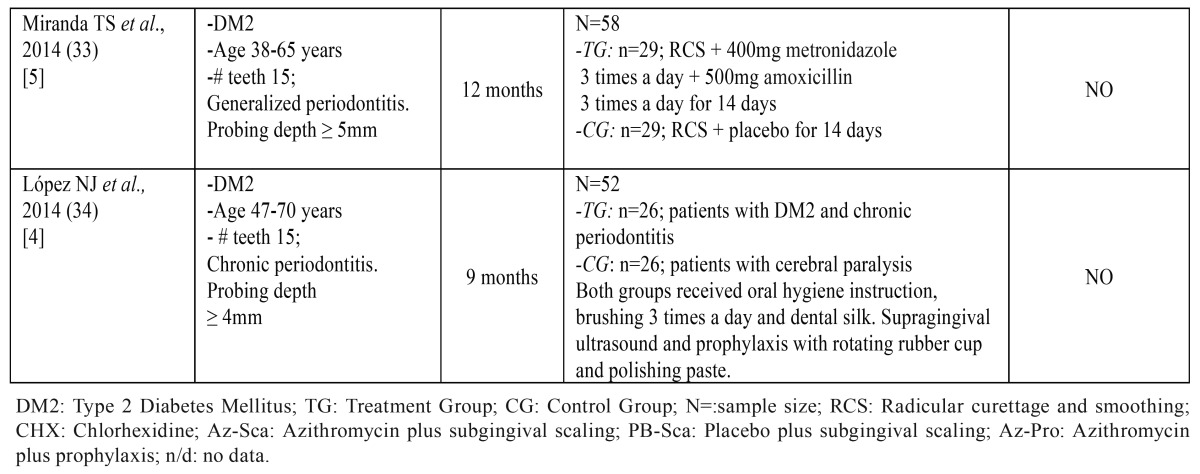
Key characteristics of the studies reviewed.

The 13 articles reviewed included a total of 1,912 patients. In three of the works, the sample was of less than 40 patients (representing 108 patients) (27,31,32) and in the other ten patient samples included more than 40 patients (representing 1,804 patients) (26,28-30,33-38) ( Table 1 and  Table 1continue).

Methodological quality evaluation found that only one article achieved a Jadad score of 5 (33); six articles obtained Jadad scores of 4 (26,30,31,34,35,37), three a score of 3 (27,28,36), two a score of 2 (29,32) and a single article scored 1 (38) ( Table 1 and  Table 1continue). 

Seven of the works reviewed (representing 998 patients; 52.3% of the 1,912 patients studied), observed a statistically significant decrease in HbA1c (*p*<0.05) (28-32,35,37). In the remaining six articles (representing 914 patients, 47.8% of the total sample), the decrease in HbA1c did not prove statistically significant (26,27,33,34,36). Patient follow-up periods across the studies varied between three and twelve months, during which, periodontal parameters, metabolic management and the treatment established in each group were evaluated. Follow-up periods were of three months (29,32,35), four months (36-38), six months (26,28), nine months (30,31,34) or twelve months (27,33).

## Discussion

The quality of the 13 articles was assessed using the Jadad scale (25) in order to reduce the risk of bias ( Table 1 and  Table 1 continue). None of the studies reviewed (26-38) had considered their level of quality, although a systematic review by Mauri *et al.*(39) did apply the same criterion to the articles analyzed. The Jadad scale awards a maximum score of 5. The present review obtained very similar results to Mauri *et al.*(39) regarding randomized trials: only one study by Miranda *et al.* (33) achieved a score of 5; six works scored 4 (26,30,31,34,35,37); six under 3. Mauri *et al.* (39) review another eight non-randomized studies, obtained one work with a Jadad score of 2, three scoring 1, and four scoring 0.

Various studies identified significant decreases in HbA1c values (28-32,35,37), although periodontal treatment, sample size, follow-up period, and quality assessment method were different in each study.

Telgi *et al.* (35), with a Jadad score of 4, a sample size of 60 patients diagnosed with moderate periodontitis, three groups, and a relatively short follow-up period of three months, found a significant decrease in HbA1c values in the two treatment groups (*p*<0.001). Both groups used a 0.12% chlorhexidine mouthwash once a day, with tooth brushing twice daily (CG), while one group was also treated with radicular curettage and smoothing (RCS). A control group used tooth brushing alone, which failed to produce a statistically significant effect (*p*<0,009).

In 2013, Engebrestson *et al.* (28) studied a large sample of 600 patients [Jadad score of 3], with a six-month follow-up, also finding a significant decrease in metabolic values in the RCS group compared with the CG group in which treatment was postponed until the end of the six-month follow-up.

Raman *et al.* (32) performed a clinical study of 32 patients divided into two groups: TG (RCS with 0.12% chlorhexidine mouthwash for 14 days) and CG (oral hygiene instruction), with a three-month follow-up. These authors also obtained a significant improvement in metabolic control.

Saengtipbovn *et al.* (29) in a study [Jadad score of 2] of 113 patients aged over 60 years, also with three-month follow-up, also obtained an improvement in metabolic control although the study did not provide a specific periodontal treatment but instead patients received lifestyle counseling and specially targeted oral hygiene instruction by means of a video aimed at proving hygiene techniques.

In studies that administered antimicrobials, such as Gaikwad *et al.* (37), [Jadad score of 4], who treated 50 patients with a four-month follow-up, also identified a significant improvement both in a group that underwent curettage and was administered doxycycline (TG), and in the control group (CG) that only underwent curettage.

Botero *et al.* (30), [Jadad 4] produced findings that were similar in some aspects. This study had a sample of 105 patients, who were treated with 500 mg azithromycin and monitored during a nine-month follow-up. This interesting study divided the sample into three groups: Group A (AZ-Sca, 500mg azithromycin per day for three days + subgingival scaling); Group B (PB-Sca, 500mg placebo per day for three days + subgingival scaling); and Group C (AZ-Pro, 500mg azithromycin per day for three days + supragingival prophylaxis). So Groups A and C received azithromycin, with Group A receiving subgingival scaling (AZ-Sca) and Group C supragingival prophylaxis (AZ-Pro). The authors found significant improvement in the patients who received the antibiotic and underwent scaling (AZ-Sca). Group B (PB-Sca, placebo + subgingival scaling) did not undergo any improvement in metabolic control.

The last in this set of studies, Pradeep *et al.* (31), [Jadad score of 4], studied 38 patients with a nine-month follow-up, dividing the sample into a treatment group (radicular curettage and smoothing plus 1.2% simvastatin [as some authors have commented that it favors bone formation]) and a control group (CG) treated with RCS and a placebo. These authors also found a significant improvement in metabolic control.

As for studies that failed to find a positive relation between periodontal treatment and metabolic control, Engebretson *et al.* (26), performed a study of 514 patients (Jadad score of 4) divided into two groups. One group postponed periodontal treatment by six months (CG), finding no improvement in metabolic control compared with the treatment group (TG) who received RCS (both manual and ultrasonic) plus chlorhexidine at the time of periodontal treatment, at three months, and at six months.

Another 2013 study by Santos *et al.* (27), [Jadad score of 3], had a small sample of 38 patients but a long follow-up of 12 months; these authors did not find a significant reduction in HbA1c, in either of the two groups, one treated with curettage and the other with curettage plus chlorhexidine.

The same results were obtained by Gay *et al.* (36) and Koromatzos *et al.* (38) [with Jadad scores of 3 and 1 respectively] in their studies. Both included samples of 126 patients each, and a follow-up of four months, establishing treatment groups (TG) who underwent RCS and control groups (CG) who received oral hygiene instruction.

Another study by Miranda *et al.* (33) [Jadad score of 5], with a sample of 58 patients and a long follow-up of 12 months, failed to find a significant decrease in HbA1c in either of its two groups, TG (RSC + 500mg amoxicillin three times a day for 14 days) and CG (RCS + placebo for 14 days).

Lastly, López *et al.* (34), [Jadad score of 4] studied a sample of 52 patients with a nine-month follow-up, divided into TG (DM2 patients with chronic periodontitis) and CG (patients with cerebral paralysis). Both groups received the same treatment by means of dental prophylaxis (oral hygiene instruction and dental cleaning three times a day using dental floss, supragingival ultrasonic descaling, and prophylaxis by rotating rubber cup and polishing paste) but no improvement in metabolic control was found.

Coinciding with those authors who claim the absence of a significant relation between periodontal treatment and metabolic control, a 2014 meta-analysis by Wang *et al.* (40) analyzed four clinical trials with 71 patients treated with oral doxycycline plus RCS and 72 patients treated with RCS and a placebo, or RCS alone. The authors conclude that metabolic control did not improve in any of the groups.

Finally, the main limitation of the research consists in the fact that high sored studies defend a relationship or no-relationship, as well a wide range of inclusion criteria, a different follow up time and a broad sample variability, making very difficult the results analysis.

## Conclusions

Most clinical studies show that radicular curettage and smoothing, whether in combination with antibiotics or not, improve the periodontal state of patients with type 2 diabetes mellitus. However, the improvement in the metabolic control through periodontal treatment is supported by seven of the reviewed articles and not validated by the rest of the articles (six).

Multicenter studies with larger patient samples and longer follow-up periods are needed to obtain results of greater meaning.

On the basis of the present literature review, the question “Does periodontal treatment influence the metabolic management of DM2?” It is still without a clear answer and there can be no definitive conclusion regarding the relation between periodontal treatment and improved glycemic control of DM2 patients.
